# Supplementary material for: A Global Screen for Assembly State Changes of the Mitotic Proteome by SEC-SWATH-MS
Source: Cell Syst. 2020 Feb 26;10(2):133–155.e6. doi: 10.1016/j.cels.2020.01.001 (PMC7042714; doi:10.1016/j.cels.2020.01.001)

Q5W111 | SPRY7_HUMAN | SPRYD7 C13orf1 CLLD6
Monomer MW [kDa]: 21.666 Monomer expected elution fraction: 54

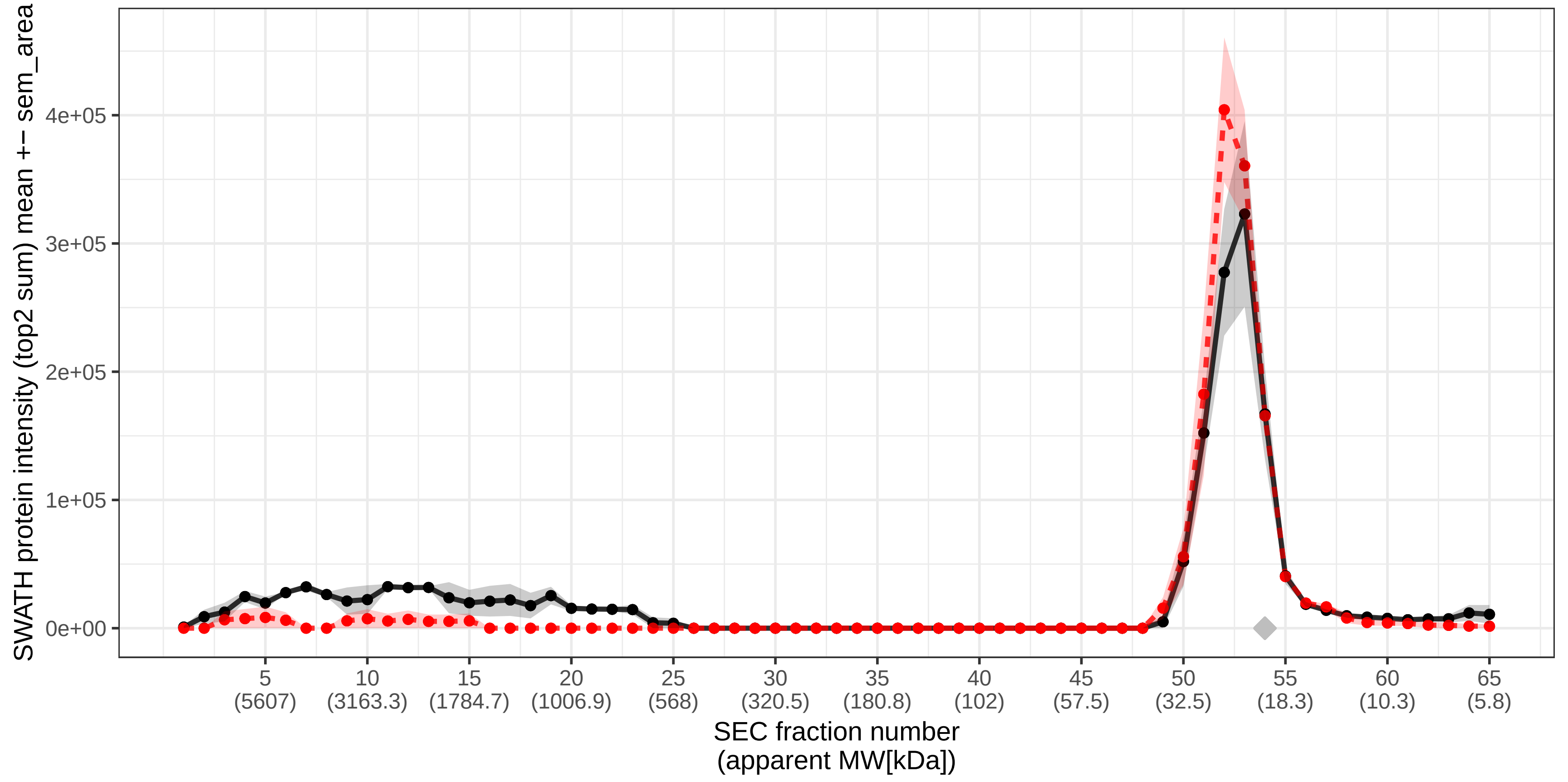

Supplement: Data S1. SEC-SWATH-MS Protein Chromatograms, Related to Figure 1 [file mmc6.zip › SECchrom_Q5W111_SPRY7_HUMAN_SPRYD7_C13orf1_CLLD6.pdf]
